# Phenolic profiles and antioxidant activity in different organs of *Sinopodophyllum hexandrum*

**DOI:** 10.3389/fpls.2022.1037582

**Published:** 2022-11-09

**Authors:** Wei Liu, Zheng Zhang, Tong Zhang, Qi Qiao, Xiaogai Hou

**Affiliations:** College of Agriculture, Henan University of Science and Technology, Luoyang, China

**Keywords:** *sinopodophyllum hexandrum*, phenols, flavonoids, phenolic acids, antioxidant activity

## Abstract

*Sinopodophyllum hexandrum* is a perennial anti-cancer medicinal plant as unique phytochemical composition podophyllotoxin, and it has special effects on the treatments of pneumonic, cervical and testicular cancers. Besides the podophyllotoxin, phenolic substances play a key role in the clinical practice. However, few reports were available in terms of the phenolic compositions and antioxidant activity. In this work, main phenolic compounds were quantified by RP-HPLC in seven organs from *S. hexandrum*. Simultaneously, the sodium borohydride/chloranil-based (SBC) method and the Folin-Ciocalteau colorimetric method were used to determine total flavonoids and total phenols contents, respectively. The antioxidant activity of the different organs was further assessed by three methods (DPPH method, ABTS method and FRAP method). Phenolic compositions/total flavonoids contents/total phenols contents/antioxidant activity was observed to have significant differences among different organs (*P*<0.05), but have a consistent changing rule viz. rhizome>root>fruit>flower>leaf>stem>petiole. Furthermore, a correlation analysis was employed and indicated that a positive correlation existed between phenolic compositions contents and antioxidant activity. Obviously, rhizome had high phenolic compositions contents and strong antioxidant activity with the low DPPH_IC50_ value of 23.52 μg/mL, high ABTS value of 1137.82 μmol equiv. Trolox/g and high FRAP value of 685.76 μmol equiv. Trolox/g. Therefore, rhizome is recommended as a preponderant medicinal part, and root is proposed as an alternative raw material resource for natural antioxidant agents in functional food, medicine and chemicals. This study can provide a new insight into the utilization extension of *S. hexandrum* resources.

## Introduction

*Sinopodophyllum hexandrum*, a sole species of *Sinopodophyllum* genus in Berberidaceae family, is an anti-cancer medicinal plant that could cure the pneumonic, cervical and testicular cancers. It is also applied in the treatment of neuroblastoma, hepatoma and leukemias (Lv and Xu, 2011; Lau and Sattely, 2015). The pharmacological foundation of *S. hexandrum* depends on aryltetralin lignans especially podophyllotoxin. Podophyllotoxin is a natural precursor for semi-synthetic production of anticancer chemotherapies including etoposide (VP-16) and teniposide (VM-26) (Canela et al., 2000; Giri and Narasu, 2000). In fact, phenolic compositions also play an important role as effective ingredients in the clinical practice. There is a growing interest in identifying new natural sources of potential medical compounds. Plant could provide a large number of natural products with diverse effects and few negative influences (Piątczak et al., 2021). Polyphenols are one good example in phytochemicals, which has broad uses. Phenolic compounds are ubiquitous secondary metabolites in the growth process of the plant, and they are helpful in beckoning pollinators, seed diffusion and diseases and pests defense (Cheynier, 2012). Plant phenol includes monophenol, diphenyl phenol, and polyphenol. Plant polyphenols are beneficial for human health, occupying relatively high proportion in phenol substances. Plant polyphenols are phenylpropanoid derivatives, including flavonoids, phenolic acids, stilbenes, and curcumins (Quideau et al., 2011). These compounds display a variety of biological activities such as antioxidation, antimicrobial action, anti-inflammation, anti-tumor and anti-virus (Maleki et al., 2019), possessing a great application potential in the sectors of drugs, foods, cosmetics, and chemicals (Yahfoufi et al., 2018; Fraga et al., 2019).

Phenolic compounds synthesised in the plant cells, and are usually known as functional ingredients as the hydrogen atoms on the aromatic ring with the hydroxyl (Alu’datt et al., 2017). Their antioxidant capacity is crucial in mitigating the negative effects of oxidative stress, which is associated with the pathogenesis of many diseases (Ma et al., 2019). These substances can be commonly divided into two main clusters, of flavonoid (flavanols, flavonols, anthocyanins) and non-flavonoid (phenolic acids, stilbenes, tannins, and their derivatives) (Zhang and Tsao, 2016; Alu’datt et al., 2017). Regard this point, the nutritious values and biological activity of phenolic compounds have been confirmed from some crops, medicinal plants and edible plants (Neri-Numa et al., 2020; de Araújo et al., 2021). Considerable epidemiological evidence indicating that the consumption of fruits and vegetables is associated with a reduction of chronic diseases such as cardiovascular diseases, neurodegeneration and some cancers (Del Rio et al., 2013). Indeed in the daily diet, the vegetable such as tomato contains abundant nutrients and chemical compositions, including minerals, vitamins C/E, carotenoids, anthocyanin, (poly)phenols, and organic acids (Martínez-Huélamo et al., 2016; Asensio et al., 2019; Cruz-Carrión et al., 2022). These bioactive compositions have to reach a target tissue in an effective concentration for their beneficial health effect (Martínez-Huélamo et al., 2016). The polyphenolic compositions are not only genotype-dependent but also they are modulated by many agronomic, geographical and seasonal factors (Cruz-Carrión et al., 2021).

Although the rhizome and root of *S. hexandrum* are the main sources of medicinal ingredients, the aerial parts (leaf, petiole, stem, flower, fruit) may also possess important pharmaceutical value. A good example is the fruit of *S. hexandrum*, which is commonly regarded as a traditional Chinese medicine (TCM) in China. In this regard, Liu et al. (2021) observed that the aerial parts (fruit, flower, leaf) of *S. hexandrum* possessed high lignans with powerful antimicrobial and cytotoxic activities, which was second only to root. It can be suggested as a potential resource for natural antimicrobial agents and cancer cell inhibitors. Lots of reports focused more on lignans not than phenolic substances, and available data on phenols are very limited in *S. hexandrum*. Despite these findings, the reference is still poor in the terms of phytochemical profile of different parts of *S. hexandrum*. In particular, there are very few studies on phenols in different parts of *S. hexandrum*. On the other hand, plant secondary metabolism is an integrative result of the interaction between plants and the environment during the long evolutionary process. The biosynthesis pathway, distribution and contents of secondary metabolites usually has the specificity as the differences in the species, organ and growth stage (Li et al., 2017). Antioxidant activity level is correlated with the type and content of chemical compositions, which is receiving increasing attention. However, antioxidant activity of the different organs of this species has not been investigated up to now. Therefore, a study on phenolic compositions and antioxidant activity is very essential in different plant organs of *S. hexandrumn* for its innovative development and utilization.

Hence, seven different plant organs of *S. hexandrum* (rhizome, root, stem, leaf, petiole, flower and fruits) were used as experimental materials, and their phenolic profiles (flavonoid, phenolic acids) were investigated by RP-HPLC in this study. The three different measurement methods (DPPH method, ABTS method, FRAP method) were simultaneously employed for antioxidant activity evaluation of seven different organs. Finally, a correlation analysis was performed between phenolic compositions and antioxidant activity. The present study aims to (1) reveal the differences in phenolic compositions among different organs, (2) antioxidant activity variations among different organs, (3) confirm the correlation between phenolic compositions content and antioxidant capacity, (4) explore an optimal part owning both high phenolic contents and strong antioxidant activity, (5) provide a new insight into the utilization expansion of *S. hexandrum* resources.

## Material and methods

### Main chemicals and instruments

Folin-Ciocalteus’s phenol reagent was procured from Beijing Solarbio Co. Ltd, PR China. 1, 1-diphenyl-2-picrylhydrazyl (DPPH), 2, 2-azino-bis (3-ethyl-benzothiazoline-6-sulphonic acid) diammonium salt (ABTS), 2, 4, 6-Tripyridyl-s-triazine (TPTZ), 6-Hydroxy-2, 5, 7, 8-tetramethylchroman-2-carboxylic acid (Trolox), and seven chemical standards (rutin, quercetin, kaempferol, gallic acid, chlorogenic acid, protocatechuic acid, ferulic acid) were provided by Sigma-Aldrich Co., St. Louis, Missouri, USA. Acetic acid, chromatographic-scale methanol and acetonitrile were obtained from Tianjin Bodi Chemical Holding Co. Ltd., China. All solutions involved in this work were filtered with a 0.22 mm nylon filter before use. Analytical-grade reagents were dissolved using deionized water (18 MΩcm). T-400B high-speed multi-function pulverizer was acquired from Dingshuai Hardware Products Co., Ltd., Yongkang City, Zhejiang Province. R-1001VN rotary evaporator was provided from Changcheng Science and Industry Co., Ltd., Zhengzhou City, Henan Province. The HPLC was carried out by an Agilent Series 1260 liquid chromatograph equipped with a quaternary gradient pump system and variable-wavelength detector (VWD) system with a reversed-phase (RP) SB-C18 column (5 μm, 4.6 ×250 mm, Agilent Technologies Inc., USA). Data collection was performed using ChemStation (Agilent Technologies Inc., USA).

### Plant materials

Plant materials were secured from *S. hexandrum* Germplasm Resource Repository of Henan University of Science and Technology (HaUST) locating in Luoyang city of Henan province in China (E112°25′23″, N34°35’46”) during April and August in the year 2020. Considered the plant age could influence the contents of phytochemicals, *S. hexandrum* with a growth age of 5 years was selected as experimental materials. In detail, stem, leaf, petiole and flower were sampled in April. The rhizome, root (namely lateral roots/fibril root) and fruit samples were collected in August when the fruits were mature stage. All fresh organs samples were placed in the shade at room temperature for natural drought. These samples were pulverized and sieved through a 40-mesh sieve, and the powder obtained was stored for further experiments. Plant material was authenticated by Ph.D. Xian Xue from HaUST. The voucher specimens of plant materials were deposited into the Herbarium/Plant Sample Collection Center of HaUST (HaUST-2020-LW000022-24).

### Preparation of the ethanolic extract for plant samples

Each powdered sample was treated using previously described methods with appropriate modification (Liu et al., 2015). In detail, plant sample extraction procedure was as following: extraction time was 1 h using extractant of 65% aqueous ethanol with liquid-solid ratio of 20:1, and extraction temperature and number of extractions is 60 °C and 3 times, respectively. The filtrates products were concentrated to obtain the crude extracts by rotary evaporation at 55 °C under the vacuum. The final extracts were stored at −20 °C in the brown bottle to avoid the light for subsequent use. All samples were performed in triplicate.

### Quantification of phenolic compositions by RP-HPLC

Test sample solution was accurately prepared using the crude extracts obtained above. Before the use, all solutions involved in this work were filtered with a 0.22 mm nylon filter. The filtered sample solution was separated to quantify the main phenolic compositions by RP-HPLC (Liu et al., 2017). Simultaneously, the validation of RP-HPLC procedure was carried out in this study. Water with 0.3% acetic acid (mobile phase A) and acetonitrile (mobile phase B) were used as mobile phases. The flow rate was set at 0.8 mL/min. The injection volume was 20 μL and the detection wavelength was 254 nm. The phenolic compositions were detected by the gradient elution program as follows: 0–20 min, 5–25% B; 20–35 min, 25–35% B; 35–40 min, 35–45% B; 40–55 min, 45–65% B, 55–60 min, 65–80% B, 60–70 min, 80–100% B; 70–75 min, 100% B. The phenols contents were calculated by referring to the internal-standard method. Each sample detection was conducted in triplicate.

### Determination of total flavonoids and total phenols contents

The total flavonoid content (TFC) was determined by the sodium borohydride/chloranil-based (SBC) method as described by He et al. (2008); Lay et al. (2014) and Mocan et al. (2015). In brief, sample solutions were diluted to a concentration of 20 mg/mL. A calibration curve was established using a series of concentration gradients of quercetin (0.1–10.0 mM). The wavelength was 430 nm for measuring the absorbance value. The TFC values were calculated as the millimoles of quercetin equivalent (QE) per 100 g dry weight (mmol QE/100 g D.W.) in *S. hexandrum*. Each determination was performed in three replicates for experimental accuracy.

Total phenol content (TPC) was quantified by the Folin-Ciocalteau colorimetric method according to Burcu et al. (2014) and Beato et al. (2011). A calibration curve was constructed *via* a batch of concentration gradients of gallic acid standard solutions (10, 20, 40, 60, 80, 100, 200, 300, and 400 μg/mL) for the TPC quantification of test samples. The absorbance was measured at 760 nm. The TPC values were expressed as the millimoles of gallic acid equivalent (GAE) per 100 g dry weight (mmol GAE/100 g D.W.). Each determination was performed in three replicates for experimental accuracy.

### Measurement of antioxidant activity

Three different methods (DPPH method, ABTS method, FRAP method) were employed to evaluate antioxidant activity of different plant organs of *S. hexandrum*. DPPH method has been widely used for the determination of antioxidant activity of pure antioxidant compounds as well as of plant natural products (Liu et al., 2016). In the present study, a DPPH method was used to investigate the DPPH radical scavenging capacity for reflecting antioxidant activity of different organs of *S. hexandrum*, as previously described by Liu et al. (2018) and Brand-Williams et al. (1995). Trolox and 80% ethanol were used as the positive and negative control, respectively. The results of DPPH radical scavenging capacity was displayed as a median inhibitory concentration, viz. DPPH_IC50_ value. The DPPH_IC50_ value is an indication of the concentration of the sample when half of the DPPH radicals are scavenged by the test sample, which is inversely proportional to antioxidant activity ability of the test samples. All measurements were carried out in triplicate.

The scavenging effects for the ABTS radical cation were investigated for all the test samples as described in previous literature (Re et al., 1999; Wang et al., 2013; Mocan et al., 2015). Standard antioxidant and blank samples were prepared with Trolox and phosphate-buffered saline (PBS) solutions, respectively. The results were expressed as micromoles of Trolox equivalents per gram dry weight (μmol equiv. Trolox/g). All measurements were repeated three times.

The ferric reducing antioxidant power (FRAP) of all samples was evaluated by the protocol described by Benzie and Strain (1996) and Wang et al. (2013). Trolox was used as the standard solution. The results of FRAP assay were displayed as micromole Trolox equivalents per gram of dry weight (μmol equiv. Trolox/g). All measurements were repeated three times.

### Statistical analysis

Correlation analysis (CA) was performed using SPSS software (SPSS for Windows 25.0, IBM SPSS Inc., Chicago, USA). Phenolic compositions were regarded as the independent variables, and antioxaidant activity was regarded as the dependent variables in CA. CA could reveal the correlation between phenos contents and antioxidant activity, providing a referenceable basis for understanding the relationship between the both.

Microsoft Excel 2016 (Microsoft Corporation, Redmond, USA) and Origin 9.0 software (OriginLab Corporation, Northampton, USA) was used for statistical analysis of the data and the related graphs creation in this study, respectively. All data was shown as mean values ± standard deviation (mean ± SD) in accordance with three independent assays (n=3). One-way analysis of variance (ANOVA) was performed using SPSS 25.0 software, based on Tukey’s HSD test at *P*<0.05.

## Results and discussion

### Validation of the HPLC procedure

Sample solutions were repeatedly injected seven times to test the precision of the RP-HPLC procedure, and plant powder samples were extracted six times for six solutions to verify the reproducibility of the procedure (Yang et al., 2007; Liu et al., 2015; Liu et al., 2016; Liu et al., 2021). The relative standard deviation (RSDs) values of relative retention times (RRTs) and relative peak areas (RPAs) were 0.023% to 0.081% and 0.165% to 2.668%, respectively, for the phenolic components of the seven replicate injections (n=7). Six replicate solid powder samples with RSDs of 0.032~0.169% and 1.328~3.163% were determined for RRTs and RPAs, respectively (n=6). Quantitative test samples were added together with known amounts of standards to confirm the accuracy of the method through a recovery experiment. The average recovery ratio of all test compositions ranged from 95.327 ± 0.453% to 101.225 ± 0.537%, with the RSDs values of 1.127%~1.783% (n=6). The limit of detection (LOD) and limit of quantification (LOQ) were the sample concentrations at a signal-to-noise ratio of 3:1 and a signal-to-noise ratio of 10:1, respectively. The LOD and LOQ of the seven compounds ranged from 0.531 to 4.225 ng/mL and 6.119 to 11.386 ng/mL, respectively. The stability of these compounds was investigated by measuring their RRTs and RPAs after the sample solutions were kept from 0 to 48 h. The RSDs of the RRTs and RPAs were found to be less than 3%. The RP-HPLC analytical procedure was proved to be reliable for the quantification of phenolic components in this study through a battery of analysises on precision, reproducibility, recovery ratio, stability, LOD and LOQ.

### Difference in flavonids and phenolic acids compounds contents in *S. hexandrum* organs

Although the substance foundation is lignans such as podophyllotoxin for medicinal application of *S. hexandrum*, phenolic compositions also play an important synergistic role in the pharmaceutical practice (Lin et al., 2008; Zhou et al., 2008). Phenolic compositions were detected using RP-HPLC method in different organs of *S. hexandrum*. The corresponding compositions were respectively identified as rutin, quercetin, kaempferol, gallic acid, chlorogenic acid, protocatechuic acid, and ferulic acid by the comparison of retention time with the standards, which was based on the Agilent ChemStation (Agilent Technologies Inc., USA). Thereinto, three substances (rutin, quercetin, kaempferol) belong to flavonoids compounds, and the remaining four substances (gallic acid, chlorogenic acid, protocatechuic acid, ferulic acid) were phenolic acids compounds.

Phenolic compositions contents had significant differences in various organs of *S. hexandrum* (*P*<0.05), and rutin and ferulic acid accounted a relatively large portion (**Table 1**). Rutin contents (0.144 ± 0.015%-0.923 ± 0.054%) and ferulic acid contents (0.147 ± 0.026%-0.834 ± 0.082%) were significantly higher than other chemical compositions among all test organs (*P*<0.05). Rutin content had the highest value of 0.923 ± 0.054% in rhizome, followed by root (0.616 ± 0.044%). Ferulic acid displayed a similar changing regulation, viz. the highest ferulic acid contents (0.834 ± 0.082%) were also found in rhizome, followed by root (0.583 ± 0.035%). In detail, rutin contents ranked in the sequence as rhizome (0.923 ± 0.054%)>root (0.616 ± 0.044%)>fruit (0.377 ± 0.053%)>flower (0.331 ± 0.035%)>leaf (0.230 ± 0.024%)>stem (0.181 ± 0.045%)>petiole (0.144 ± 0.015%) in different plant organs. Other substance distribution pattern was consisitent with the rutin among different plant organs. For example, quercetin content ranked in this order as rhizome (0.399 ± 0.028%)>root (0.318 ± 0.017%) > fruit (0.237 ± 0.030%)>flower (0.194 ± 0.026%)>leaf (0.157 ± 0.023%)>stem (0.136 ± 0.006%)>petiole (0.100 ± 0.005%). The previous reports have investigated the active ingredients including flavonids and phenolic acids in different *S. hexandrum* organs, and showed that active ingredients contents significantly changed along with different organs (*P*<0.05), always reaching a peak value in the rhizome followed by root (Liu et al., 2021). Indeed, the results were agreement with the present studies.

**Table 1:** The contents of phenols including flavonids and phenolic acids in different organs of *S. hexandrum*.

Code	Organ	Flavonids	Phenolic acids					
		Rutin (%)	Quercetin (%)	Kaempferol (%)	Gallic acid (%)	Chlorogenic acid (%)	Protocatechuic acid (%)	Ferulic acid (%)
1	Rhizome	0.923 ± 0.054a	0.399 ± 0.028a	0.293 ± 0.016a	0.323 ± 0.074a	0.171 ± 0.018a	0.189 ± 0.038a	0.834 ± 0.082a
2	Root	0.616 ± 0.044b	0.318 ± 0.017b	0.229 ± 0.017b	0.258 ± 0.066b	0.136 ± 0.010b	0.157 ± 0.018b	0.583 ± 0.035b
3	Stem	0.181 ± 0.045f	0.136 ± 0.006e	0.102 ± 0.007e	0.118 ± 0.008e	0.086 ± 0.005d	0.106 ± 0.005d	0.228 ± 0.031f
4	Petiole	0.144 ± 0.015g	0.100 ± 0.005f	0.091 ± 0.004e	0.089 ± 0.008f	0.077 ± 0.004e	0.087 ± 0.005e	0.147 ± 0.026g
5	Leaf	0.230 ± 0.024e	0.157 ± 0.023e	0.116 ± 0.011d	0.137 ± 0.006d	0.098 ± 0.006d	0.118 ± 0.007d	0.289 ± 0.016e
6	Flower	0.331 ± 0.035d	0.194 ± 0.026d	0.131 ± 0.007d	0.190 ± 0.036c	0.114 ± 0.008c	0.129 ± 0.005c	0.405 ± 0.053d
7	Fruit	0.377 ± 0.053c	0.237 ± 0.030c	0.173 ± 0.028c	0.208 ± 0.044c	0.128 ± 0.009b	0.137 ± 0.008c	0.486 ± 0.041c

All data is denoted as mean ± SD (n=3). Significant differences between different treatments (samples) in the same column are indicated by different lowercase letters (P<0.05).

In addition in *S. hexandrum*, the phenomenon of chemical compositions contents varying with plant organs was also observed in other chemical ingredients types such as lignans, podophyllotoxin and 4’-demethylpodophyllotoxin (Li et al., 2015; Li et al., 2017), as well as nutritional ingredients such as soluble sugar and trace metal elements (Ma et al., 2002). Similar result was also verified in its sibling species *Podophyllum peltatum* (Yin et al., 2021), *Dysosma versipellis* (Deng et al., 2006; Ma et al., 2014), and *D. tsayuensis* (Zhong et al., 2011). These results consistently demonstrated that observable diversities existed in chemical compositions of different organs of *S. hexandrum*. In different plant organs, chemical compositions distribution variation is an integrative reflect of reproductive strategy as well as biochemical-ecological adaptation of the plant-environment (Ma et al., 2002; Lu et al., 2006), likely depending on growth period, sampling time, experimental methods and environmental factors.

Rutin, quercetin, kaempferol, gallic acid, chlorogenic acid, protocatechuic acid, and ferulic acid are beneficial for human health maintenance (Harwood et al., 2007; Wang et al., 2013). Previous studies have demonstrated that *S. hexandrum* contains high levels of these active components, which could be regarded as natural antioxidants with significant application potential for use in the pharmaceutical, food and chemical industries (Lin et al., 2008). Phenolic compounds are synthesized by the pentose phosphate pathway (PPP), shikimate pathway, and phenylpropanoid pathway (Randhir et al., 2004). The first rate-limiting enzyme is glucose-6-phosphate dehydrogenase (G6PDH) for the phenols synthesis in PPP (Liu et al., 2013). Phenylalanine ammonia-lyase (PAL) is a key enzyme in the phenylpropanoid pathway (González-Chavira et al., 2018). PAL can change many chemical compositions structure, such as ferulic acid and caffeic acid, facilitating the phenols production (Jeong et al., 2018; Lyu et al., 2022). Also, Liu et al. (2013) and Ren & Sun (2014) have verified that the enzyme activity increase of PAL and G6PDH can improve the phenolics contents. Subsequently, molecular mechanism of phenolic compositions conetents difference should be performed in different organs of *S. hexandrum* at molecular level. In summary, the contents of the phenols are relatively high and vary considerably in different organs of *S. hexandrum*. A more comprehensive investigation of phenolic constituents in *S. hexandrum* appears essential for its effective quality evaluation.

### Difference in total flavonoids contents and in total phenols contents

In this study, the total flavonoids contents and total phenols contents were also investigated for an integrative comparative purpose of different organs in *S. hexandrum*. The results were displayed in **Table 2**. The results showed that the total flavonoid contents and total phenol contents significantly varied with different plant organs. In different organs of *S. hexandrum*, total flavonids contents ranged from 11.68 ± 0.59 to 27.24 ± 2.12 mmol QE/100 g D.W., and 33.26 ± 1.19-67.86 ± 3.11 mmol GAE/100 g D.W. for total phenols contents. Total phenols contents were more abundant than total flavonoids. The highest total phenols contents were detected in rhizome with the contents of 67.86 ± 3.11 mmol GAE/100 g D.W., followed by root with the contents of 61.35 ± 3.02 mmol GAE/100 g D.W., and the lowest was in petiole with the contents of 33.26 ± 1.19 mmol GAE/100 g D.W. Consistent with results of total phenols contents, the highest total flavonids contents were observed in rhizome with the contents of 27.24 ± 2.12 mmol QE/100 g D.W., followed by root with the contents of 22.16 ± 1.16 mmol QE/100 g D.W., and the lowest was in petiole with the contents of 11.68 ± 0.59 mmol QE/100 g D.W. Obviously, there were significant differences in total flavonoids and total phenols contents in various *S. hexandrum* organs. Furthermore, effective ingredients content difference is likely to lead to different bioactivities and therapeutic effect of various parts from the same species. However, few reports were availabe in the aspects of total flavonoid and total phenols contents of *S. hexandrum* organs. Li et al. (2017) investigated inflence of the altitude on total flavonoid and total phenols contents of *Podophyllum hexandrum* (syn. *Sionpodophyllum hexandrum*) using different altitude heights (2400-2500 m and 2900-3000 m), and found low altitude is conducive for fruit growth and development and for the accumulation of total flavonoid and total phenols in the fruit; meanwhile, the results indicated that total flavonoids and total phenols contents in the fruit peel were higher than those in fruit pulp in the same altitude.

**Table 2:** Total flavonoids contents in different organs of *S. hexandrum* as well as total phenols contents.

Code	Organ	Total flavonoids contents (TFC) (mmol QE/100 g D.W.)	Total phenols contents (TPC) (mmol GAE/100 g D.W.)
1	Rhizome	27.24 ± 2.12a	67.86 ± 3.11a
2	Root	22.16 ± 1.16b	61.35 ± 4.02b
3	Stem	14.66 ± 0.76d	38.43 ± 2.57d
4	Petiole	11.68 ± 0.59e	33.26 ± 1.19e
5	Leaf	14.82 ± 0.88d	41.55 ± 2.33d
6	Flower	17.27 ± 1.02c	49.68 ± 2.31c
7	Fruit	19.58 ± 1.03c	52.51 ± 3.15c

All data is presented as mean ± SD (n=3). Significant differences between different treatments (samples) in the same column are indicated by different lowercase letters (P<0.05).

In summary, in all test organs of *S. hexandrum*, total flavonoid contents had the same ranking order as rhizome>root>fruit>flower>leaf>stem>petiole with total phenols contents. Therefore, among the various *S. hexandrum* organs, rhizome was the best part because of its abundant flavonoid and phenol contents, followed by root. The distribution pattern of the total flavonoids and total phenols contents in various *S. hexandrum* organs were likely due to the differences in the types, proportions, and structures of compound monomers. The observed results, in turn, may be attributed to the differentce in biosynthesis, accumulation and storage locations of compound monomers.

### Antioxidant activity of different organs of *S. hexandrum*

Free radical is an intermediate metabolite of various biochemical reactions in human life activities. It has high chemical activity and is an effective defense system of the human-body. However, excessive accumulation of free radical that is unable to be scavenged in time would attack the life macromolecules substances and various organelles, and caused the human-boday damage in molecular, cell and tissue level, which could further accelerate the aging-process of human-boday and induced various chronic diseases (Brand-Williams et al., 1995; Akbari et al., 2022; Anand et al., 2022). As the improvement of people’s cognitive level, more and more people realized the harm of free radicals. In order to reduce the harm of free radicals, a lot of antioxidants were synthesized, but artificial synthetic antioxidants have high toxicity. For exmple, toxic and carcinogenic effects of butylated hydroxyanisole (BHA), butylated hydroxy toluene (BHT) and tertiary butylhydroquinone (TBHQ) have been confirmed in the mice and other biological organisms (Goh et al., 2003; Mira-Sánchez et al., 2019). Therefore, antioxidant activity should be evaluated in different plant organs of *S. hexandrum*, which is essential and meaningful to develop the natural antioxidants for functional foods, medicines and health-care products.

**Figure 1:**
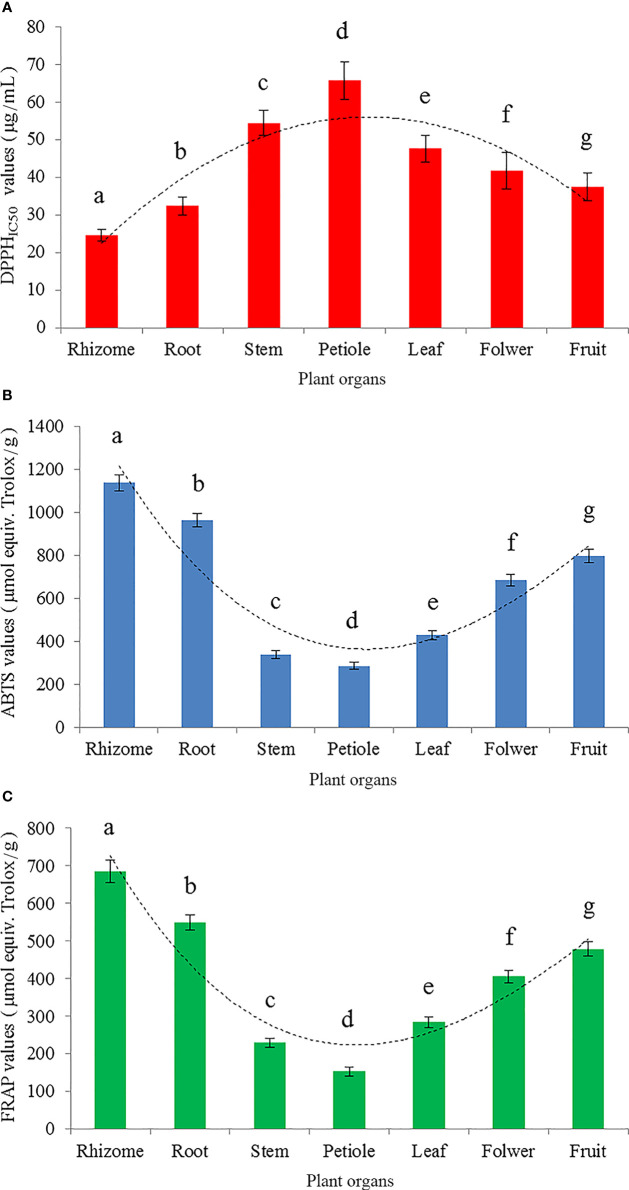
Antioxidant activity represented by DPPH_IC50_ **(A)**, ABTS **(B)** and FRAP **(C)** for different organs of *S. hexandrum*. DPPH_IC50_ value is an effective sample concentration at which DPPH radicals were scavenged by 50%. ABTS and FRAP values can reflect the results of ABTS radical cation scavenging assay and ferric reducing power assay, respectively. ABTS and FRAP values are both expressed as micromoles of trolox equivalent per gram. All data is presented as mean ± SD (n=3). Different lowercase letters indicate the significant difference by ANOVA with Tukey’s multiple range test (P<0.05).

Three different antioxidant activity assessment methods (DPPH method, ABTS method and FRAP method) were used to measure the antioxidant activity of different organs of *S. hexandrum* (**Figure 1**). The results of the DPPH method, ABTS method and FRAP method were respectively presented in **Figures 1A–C** using DPPH_IC50_, ABTS and FRAP values. In detail, the DPPH_IC50_ values showed a parabolic change trend which firstly increased and then decreased from rhizome to fruit, and peaked at the petiole. DPPH_IC50_ value, a median-inhibitory concentration, is a sample concentration at which the clearance rate reached 50%. The smaller DPPH_IC50_ value indicates the test sample has the stronger antioxidant activity. The DPPH_IC50_ values ranked as petiole (64.75 μg/mL)>stem (53.39 μg/mL)>leaf (46.55 μg/mL)>flower (40.68 μg/mL)>fruit (36.44 μg/mL)>root (31.37 μg/mL)>rhizome (23.52 μg/mL) (**Figure 1A**), whereas antioxidant activity ability is ranked in the opposite order, viz. rhizomes>root>fruit>flower>leaf>stem>petiole. Specially, the petiole had the largest DPPH_IC50_ value, and was 2.75 times as rhizomes with the smallest DPPH_IC50_ value. Therefore, the rhizome possessed the strongest antioxidant activity, and the petiole was the weakest among the seven different test organs. Additionally, DPPH_IC50_ value of positive control trolox was 9.41 μg/mL. *S. hexandrum* rhizome has relatively strong antioxidant activity compared with positive control, which accounted for 40% of trolox.

The other two evaluation methods, ABTS method (**Figure 1B**) and FRAP method (**Figure 1C**), had the consistent results, which agreed with DPPH method. In brief, the results from three different evaluation methods consistently showed that the antioxidant activity was significantly different among different organs of *S. hexandrum* (*P*<0.05), but the changing regulation of the antioxidant activity ability was observed as the same sequence of rhizome>root>fruit>flower>leaf>stem>petiole (**Figures 1A–C**). Obviously, rhizome and root possessed relatively high antioxidant activity, and could be used as the predominate raw materials for natural antioxidants production.

### Correlation analysis between the phenolic profiles contents and antioxidant activity

A correlation analysis was conducted to further investigate the relationship between the phenolic profiles contents and antioxidant activity using SPSS25.0 software. The range of correlation coefficients was 0.533-0.959 (**Table 3**). The results showed that antioxidant activity had a positive correlation with phenolic constituents contents at different levels. In flavonoids compositions, rutin contents had high correlation with DPPH_IC50_/ABTS/FRAP values (0.652-0.767), and was significantly and positively correlated with ABTS/FRAP values (P<0.05). In phenolic acids, chlorogenic acid contents had high correlation with DPPH_IC50_/ABTS/FRAP values (0.685-0.882). Chlorogenic acid content was significantly and positively correlated with ABTS/FRAP values (P<0.05), and a high significant correlation was found between chlorogenic acid contents and ABTS values (P<0.01). It’s worth noting that the total flavonoids contents and total phenols contents had high positive correlation with DPPH_IC50_/ABTS/FRAP values (0.847-0.959) (P<0.01), and an extremely significant and positive correlation existed between total flavonoids contents and ABTS values (0.935) (P<0.001). Total phenols contents had also extremely significant and positive correlation with DPPH_IC50_/FRAP values (P<0.001).

**Table 3:** Correlation coefficients between the phenolic profiles contents and antioxidant activity for different organs of *S. Hexandrum*.

Items	DPPH_IC50_ values	ABTS values	FRAP values
Rutin	0.652	0.731*	0.767*
Quercetin	0.533	0.675	0.733*
Kaempferol	0.729*	0.668	0.657
Gallic acid	0.647	0.708*	0.836**
Chlorogenic acid	0.685	0.882**	0.705*
Protocatechuic acid	0.721*	0.783*	0.683
Ferulic acid	0.638	0.867**	0.789*
Total flavonoids contents (TFC)	0.847**	0.935***	0.854**
Total phenols contents (TPC)	0.946***	0.859**	0.959***

A single asterisk (*) indicates significant correlation (P<0.05), a double-asterisk symbol (**) indicates highly significant correlation (P<0.01), and an asterism (***) indicates extremely significant correlation (P<0.001).

In fact, this study have found that the change regulation of phenolic composition contents (rhizome>root>fruit>flower>leaf>stem>petiole) was in conformity with the antioxidant activity in different organs of *S. hexandrum* by the investigation of phenolic compositions and antioxidant activity in aboved description (**Table 1** and **Figure 1**). Similary, the change regulation of total flavonoids contents and total phenols contents also agreed with the that of antioxidant activity (**Table 2** and **Figure 1**). Namely, the plant organ with higher chemical compositions contents also has stronger antioxidant activity, well verifying the results of correlation analysis. A positive correlation between chemical compositions and antioxidant activity had been described in previous reports (Dar et al., 2013). For example, Li et al. (2017); Li et al. (2018) found antioxidant activity of *S. hexandrum* was stronger in higher altitude areas, because where podophyllotoxin was more easily accumulated, forming high podophyllotoxin contents. The results from Wang et al. (2013) showed antioxidant activity has a positive correlation with flavonoids and phenolic compounds. Liu et al. (2020) found a similar phenomenon in different *Rehmannia glutinosa* samples, which possibly attributed to the high chemical ingredients such as catalpol (6.74 mg/g), rehmaionoside A (1.93 mg/g) and rehmannioside D (5.13 mg/g). However, such a rule has not been found in all the medicinal plants, that is, the plant organ with high chemical ingredients contents could not always have strong antioxidant activity. Liu et al. (2016) found that *Potentilla fruticosa* leaf had high chemical compositions contents (quercetin, rutin, etc.), but its antioxidant activity was low compared with other test samples. This phenomenon may have been related to the plant species type, chemical ingredients synergy, genetic factors and external environmental factors.

## Conclusions

Monomeric compounds of phenol substances, total flavonoids contents, total phenol contents and antioxidant activity were investigated in the present study, as well as the correlation between phenolic compositions contents and antioxidant activity. All resulting data demonstrated that there were significant differences in monomeric compounds, total flavonoids contents, total phenol contents and antioxidant activity among different organs of *S. hexandrum* (P<0.05). Whereas, they have consistent changing rule of rhizome>root>fruit>flower>leaf>stem>petiole. Moreover, antioxidant activity is significantly and positively correlated with phenolic compositions contents. Considering the high phenol percentages and antioxidant activity, rhizome was regarded to possess the high quality, followed by root. The results indicates that the rhizome and/or root extraction can be considered as a potential raw material source that can be used as natural antioxidant agents in the production of food, medicine, cosmetic and chemicals. In conclusion, this study well investigated the phenolics and antioxidant activity of *S. hexandrum*, providing a valuable reference for its innovative and comprehensive utilization.

In the following research, we should increase the sampling areas, collect more *S. hexandrum* samples, comprehensively evaluate their phenolic compositions, antioxidant activity and the relationship between the two. Meanwhile, biochemistry, phytochemistry, molecular biology and multi-omics method should be combined with *in vivo* antioxidant activity evaluation methods to investigate the biosynthesis and antioxidation mechanism of phenolic compounds at the gene level.

## Data availability statement

The original contributions presented in the study are included in the article/supplementary material. Further inquiries can be directed to the corresponding author.

## Author contributions

WL and XH designed the experiments and supervised the project. WL, ZZ and TZ performed the experiments. WL, ZZ and TZ analyzed the data and prepared the manuscript. QQ and XH commented and revised the manuscript. WL and XH were responsible for ensuring that the descriptions are accurate and agreed by all authors. All authors contributed to the article and approved the submitted version.

## Funding

This work was supported by the Project for Technical system of Tradiational Chinese Medicinal Material Industry in Henan Province (YuCaiKe [2022] No.24), the Program for Science and Technology Development of Henan Province (No. 222102110140) and the Project for National Natural Science Foundation of China (No. 81803659).

## Conflict of interest

The authors declare that the research was conducted in the absence of any commercial or financial relationships that could be construed as a potential conflict of interest.

## Publisher’s note

All claims expressed in this article are solely those of the authors and do not necessarily represent those of their affiliated organizations, or those of the publisher, the editors and the reviewers. Any product that may be evaluated in this article, or claim that may be made by its manufacturer, is not guaranteed or endorsed by the publisher.
